# Measles Outbreak Response Activity in Japan, and a Discussion for a Possible Strategy of Outbreak Response Using Cycle Threshold Values of Real-Time Reverse Transcription PCR for Measles Virus in Measles Elimination Settings

**DOI:** 10.3390/v15010171

**Published:** 2023-01-06

**Authors:** Junji Seto, Yoko Aoki, Kenichi Komabayashi, Keiko Yamada, Hitoshi Ishikawa, Tomoo Ichikawa, Tadayuki Ahiko, Katsumi Mizuta

**Affiliations:** 1 Yamagata Prefectural Institute of Public Health, Yamagata 990-0031, Japan; 2 Okitama Public Health Center, Yamagata 992-0012, Japan; 3 Department of Physical Therapy, Yamagata Prefectural University of Health Sciences, Yamagata 990-2212, Japan; 4 Department of Society and Regional Culture, Okinawa International University, Ginowan 901-2701, Japan; 5 Department of Health and Welfare, Yamagata Prefectural Government, Yamagata 990-8570, Japan

**Keywords:** breakthrough infection, elimination, measles virus, naïve infection, preparedness, public health response, real-time reverse transcription PCR, retrospective contact tracing, super-spreader, vaccination

## Abstract

Measles is a highly contagious, but vaccine-preventable disease caused by the measles virus (MeV). Although the administration of two doses of measles vaccines is the most effective strategy to prevent and eliminate measles, MeV continues to spread worldwide, even in 2022. In measles-eliminated countries, preparedness and response to measles outbreaks originating from imported cases are required to maintain elimination status. Under these circumstances, real-time reverse transcription (RT) PCR for MeV could provide a diagnostic method capable of strengthening the subnational capacity for outbreak responses. Real-time RT-PCR can detect MeV RNA from patients with measles at the initial symptomatic stage, which can enable rapid public health responses aimed at detecting their contacts and common sources of infection. Furthermore, low cycle threshold (*Ct*) values (i.e., high viral load) of throat swabs indicate high infectiousness in patients with measles. The high basic reproduction number of measles suggests that patients with high infectiousness can easily become super-spreaders. This opinion proposes a possible strategy of rapid and intensive responses to counter measles outbreaks caused by super-spreader candidates showing low *Ct* values in throat swabs. Our strategy would make it possible to effectively prevent further measles transmission, thereby leading to the early termination of measles outbreaks.

## 1. Introduction

Measles is one of the most highly contagious diseases (basic reproduction number R_0_ = 12–18) [1]. It is caused by the measles virus (MeV), which is a member of the *Morbillivirus* genus of the family *Paramyxoviridae* [1]. The MeV is an RNA virus with a single-stranded, negative-sense, non-segmented genome. This virus can be subdivided into 24 genotypes based on the 450-nucleotide sequence of the nucleocapsid protein, but it is antigenically monotypic [1,2]. Therefore, the attenuated measles vaccines derived from a single MeV genotype isolated in the 1950 s remain protective [1]. The administration of two doses of the measles vaccine has been used worldwide as a strategy for eliminating measles [1]. However, MeV continues to spread among humans, even today.

With the end of the COVID-19 pandemic in sight, measles is showing signs of re-emergence [3]. Globally, the number of measles cases has decreased from 873,022 in 2019 to 159,067 in 2020 [4]. The sharp drop in the number of cases since April 2020 [3], immediately after the declaration of the COVID-19 pandemic by the World Health Organization (WHO), suggests that worldwide COVID-19 countermeasures (e.g., lockdown, self-quarantine, and wearing masks) may have had a positive impact on the decline of measles cases [5]. Additionally, 2021 also demonstrated a low number of cases—124,041 [4]. However, provisional data indicate that the number of measles cases as of September 2022 has already exceeded the number of cases in 2021 [3]. From January to September 2022, 97.7% of measles cases were reported in the regions of Africa, southeast Asia, and the eastern Mediterranean, where measles elimination has not progressed sufficiently [3].

For 76 (39.2%) of the 194 countries considered to have eliminated measles as of September 2022 [3], the sustained vaccination of more than 95% of the population using quality-assured vaccines is required to maintain the elimination status and to prevent measles outbreaks caused by imported cases [1]. However, delays in measles vaccination programs due to the COVID-19 pandemic have made it difficult to sustain the status of elimination [1]. Furthermore, primary or secondary vaccine failures and/or vaccine hesitancy are inhibitory factors of population immunity [6,7]. In these situations, outbreak responses conducted by public health officials play a crucial role in maintaining the status of measles elimination [8,9,10].

Measles cases can be primarily subdivided into two clusters: (1) cases with naïve infection (naïve cases), which depend on no vaccination history or primary vaccine failure and (2) cases with breakthrough infection (breakthrough cases), which depend on secondary vaccine failure or reinfection [6,11,12]. Naïve cases show extremely high infectiousness, that is, they can be super-spreaders [13]. A typical example of transmission by naïve cases without vaccination history is the first epidemic of measles in Greenland in 1951, where all but five of 4262 theoretically susceptible individuals were infected with measles, with a mortality of 1.8% [14]. In contrast, transmission of measles from breakthrough cases, showing mild illness called modified measles, is scarce [10,11,12,15]. Indeed, a previous study showed that MeV cannot be isolated in specimens from breakthrough cases [12]. Therefore, the more the vaccination progresses, the more the proportion of patients with low infectiousness increase. In particular, in countries where a high proportion of vaccination is maintained, it is important to classify measles cases into high infectiousness (naïve cases) and low infectiousness (breakthrough cases). Laboratory diagnosis is necessary to distinguish between these cases [11,12].

Rapid and accurate laboratory diagnosis is helpful in breaking the chain of measles transmission. Internationally, Immunoglobulin M (IgM) testing is the mainstream method for the diagnosis of measles infection [16]; however, it is unsuitable for rapid diagnosis because IgM is only detectable after the appearance of a rash [1]. For example, an outbreak study targeting measles cases testing positive for MeV-RNA reported that 82.1% of serum specimens collected up to 4 days after the onset of initial symptoms were negative for IgM [9]. In contrast, MeV-RNA can be detected in clinical specimens at the onset of initial symptoms such as fever, coryza, and conjunctivitis [1]. In addition to conventional reverse transcription (RT) PCR, real-time RT-PCR, which can estimate the viral load in a specimen, is currently available for the detection of MeV-RNA [1,2,17]. However, the application of these PCR methods to measles outbreaks has not been fully discussed, even in the “Measles outbreak guide” published by the WHO in August 2022 [8].

In this opinion, we first summarize the history of measles elimination in Japan to highlight the importance of national vaccination policies. Thereafter, we propose a possible strategy for outbreak response using cycle threshold (*Ct*) values of real-time RT-PCR for MeV for identifying potential super-spreaders and effectively preventing further measles transmission in measles-eliminated countries.

## 2. History of Measles Elimination in Japan

In Japan, the term “*Akamogasa* (i.e., red maculopapular rash)”, described in three classical books in 998 (middle of the Heian period), is the first description of a measles outbreak [18]. Between 998 and 1862, 38 measles outbreaks were reported (median interval [IQR]: 21 years [12–30 years]) [18]. In approximately 1200 (the Kamakura period), measles became known as “*Hashika*” and this popular name is still used today.

An analysis of measles mortality between 1900 and 1960 showed that measles became endemic in Japan due to urbanization, the development of transportation networks, and the fact that almost all children began to attend elementary school in the 1900s owing to the policy of universal elementary education promulgated in 1900 [19].

**Figure 1:**
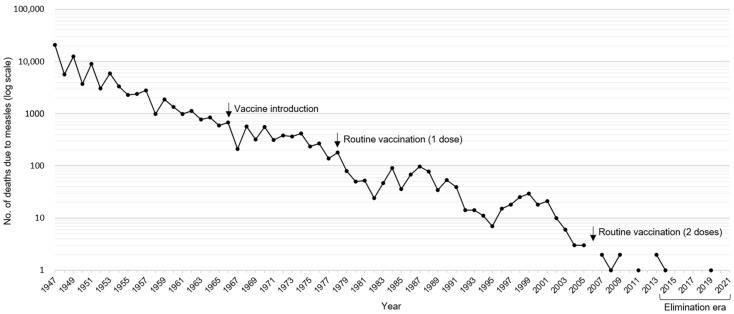
Number of measles deaths in Japan in 1947−2021 [20,21]. The number of deaths has smoothly declined, mainly because of Japan’s national vaccination policies (arrows). The vertical axis is denoted by the log scale since the number of deaths declined sharply from 20,939 in 1947 to 0 in 2021.

The number of measles deaths in Japan is shown in Figure 1 and is based on the Vital Statistics of Japan from 1947 to 2021 (1947–1949, personal records from a currently unavailable webpage; 1950–1999, referred from [20]; 2000–2021, referred from [21]). In 1947, which was the recovery period from the disruption caused by World War II, 20,939 people died from measles. Thereafter, the number of measles deaths gradually declined due to improvements in the social environment. After the introduction of the measles vaccine in 1966, the annual number of deaths decreased to several hundred. When routine measles vaccination of one dose for children began in 1978, the annual number of deaths decreased to less than 100 persons from the following year onward. In addition to the measles vaccine, the measles–mumps–rubella vaccine was introduced in 1989. However, the combined vaccine showed the problem of aseptic meningitis caused by the mumps component, and official cessation of the combined vaccine was enforced in 1993 [22]. This problem has resulted in the stagnation of vaccination strategies in Japan. In contrast, two doses of routine vaccination using the measles–rubella vaccine (MRV) were introduced in 2006, without the problem of side effects. Currently, the annual number of deaths caused by measles has ranged from 0 to 2. As such, even though measles is a potentially fatal infectious disease [1], the number of measles deaths in Japan has declined, mainly because of its national vaccination policies. In fact, the MRV coverage was 98.5% for the first dose (1–2 years of age) and 94.6% for the second dose (5–7 years of age) in 2020 [23].

In contrast to the number of deaths, the number of measles cases did not decline successfully. Approximately 200,000 measles cases were estimated from the analysis of the sentinel surveillance data in 2000 [22]. In 2001, more than 265,000 people were estimated to have suffered from measles [24]. Thereafter, the number of measles cases declined gradually. However, measles cases among 10−30-year-old individuals increased again in 2007 [24], and measles cases in Japanese youths have caused several outbreaks overseas [25,26]. Consequently, Japan was deemed as a “measles-exporting country” during this time. To overcome this situation and achieve the goal of measles elimination, the Japanese government launched a national strategy at the end of 2007. Specifically, (1) the MRV catch-up campaign for 13- and 18-year-olds was conducted during 2008–2012, (2) a shift in the reporting system from sentinel surveillance to notifiable disease surveillance (i.e., all measles cases must be reported to public health centers) started in 2008, and (3) rapid public health responses to measles cases, were conducted [24].

**Figure 2:**
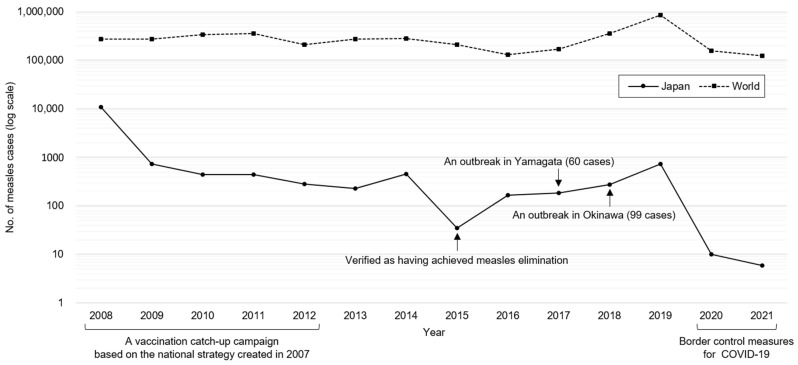
Number of measles cases worldwide and in Japan since 2008, when a notifiable disease surveillance in Japan was started, as well as relevant events in Japan [4,27]. The vertical axis is denoted by the log scale since the number of cases in Japan has declined sharply from 11,013 in 2008 to 6 in 2021. A vaccination catch-up campaign in Japan during 2008–2012 resulted in declining number of cases and Japan was verified as a country that has achieved measles elimination in 2015. Several large outbreaks have occurred thereafter [9,28], but the number of cases has decreased dramatically in 2020 and 2021 because of border control measures for COVID-19. During the period, the number of measles cases worldwide has been in the range of 124,041 (2021)–873,022 (2019) [4].

The number of measles cases worldwide and in Japan (which has a total population of approximately 126 million) and relevant events in Japan are shown in Figure 2 [4,27]. In 2008, 11,013 cases were reported due to a measles outbreak that continued from the previous year. In contrast, the number of cases remained low after 2009 due to the vaccine catch-up campaign for teenagers and the national advocacy for two doses of MRV [24]. Furthermore, the nationwide molecular epidemiology of MeV showed that D5, an endemically circulated strain in Japan, has not been detected since 2009 [2]. Consequently, Japan was verified by the WHO Western Pacific Regional Office in March 2015 as a country that has achieved measles elimination (Figure 2) [10]. However, imported cases and cases among their contacts have been continuously reported, and some of them caused outbreaks. Particularly, measles outbreaks that accounted for more than 30% of the annual number of cases occurred in 2017 and 2018 (Figure 2) [9,28]. Moreover, measles cases in Japan have increased proportionally to the rapid increase in global cases in 2019. However, the number of measles cases has decreased dramatically in 2020 and 2021 because of border control measures for COVID-19.

As of 11 October 2022, COVID-19 border control measures are virtually non-existent. Accordingly, Japan now strongly requires preparedness strategies for measles outbreaks originating from imported cases.

## 3. Outbreak Response Using *Ct* Values of Real-Time RT-PCR

### 3.1. Requirements for Accurate Real-Time RT-PCR Results

Real-time RT-PCR contributes to the rapid confirmation of acute measles cases [1]. However, this method has more stringent requirements than do serological assays in terms of sample quality, which can be influenced by collection, transportation, and storage conditions [1]. Therefore, highly accurate testing of high-quality specimens is essential for conducting outbreak responses using *Ct* values (Table 1). In measles-eliminated countries targeted in this opinion, breakthrough cases accounted for more than half of the cases [9,10,11,12]. To accurately identify these breakthrough cases by real-time RT-PCR, throat (or nasopharyngeal) swabs, peripheral blood monocular cells (PBMCs), and urine are available, but serum is unsuitable because of the extremely low positivity rate [10,11,12]. Throat swabs should be collected using a synthetic fiber swab to ensure specimen quality [29]. Furthermore, it is desirable to collect specimens as early as possible after the onset of symptoms to prevent false-negative results. Real-time RT-PCR is less affected by RNA fragmentation because it detects short complementary DNA fragments (e.g., 75 nucleotides) [1,17]. However, given the fact that breakthrough cases often show high *Ct* values (i.e., low viral load) [10,11,12], cold chain and rapid transportation are important to minimize the fragmentation of MeV-RNA [1]. Robust transportation systems can be challenging in several countries. However, considering that MeV and SARS-CoV-2 are both enveloped RNA viruses [1,30], a transportation system for COVID-19 could also be applicable to measles. The cost of robust transportation and high-quality laboratory testing (Table 2) is expected to be allocated from each country to strengthen outbreak preparedness for measles [31].

### 3.2. Outbreak Response Using Ct Values

In countries where measles has been eliminated, the rapid and accurate detection of measles cases by laboratory diagnosis can help in outbreak responses [16,31]. Here, we show a possible strategy for the outbreak response of measles in subnational regions based on our experience of an outbreak in Yamagata Prefecture, Japan, in 2017 (Figure 2). The outbreak was largest at that time of the measles elimination era, and public health centers performed health observations for approximately 3700 contacts [9], which represented 0.3% of the total population in Yamagata. Owing to this difficult experience, we investigated methodologies to reduce the burden on public health centers with limited human resources. Consequently, we highlight a methodology to estimate the infectiousness of patients with measles using the *Ct* values of throat swab specimens [9,10]. In Japan, the country used as a model for this opinion, the notification of all measles cases from physicians to public health centers has been mandatory since 2008. Furthermore, in principle, MeV-RNA testing and IgM measurement are necessary for all patients clinically diagnosed with measles.

#### 3.2.1. Routine Surveillance

**Figure 3:**
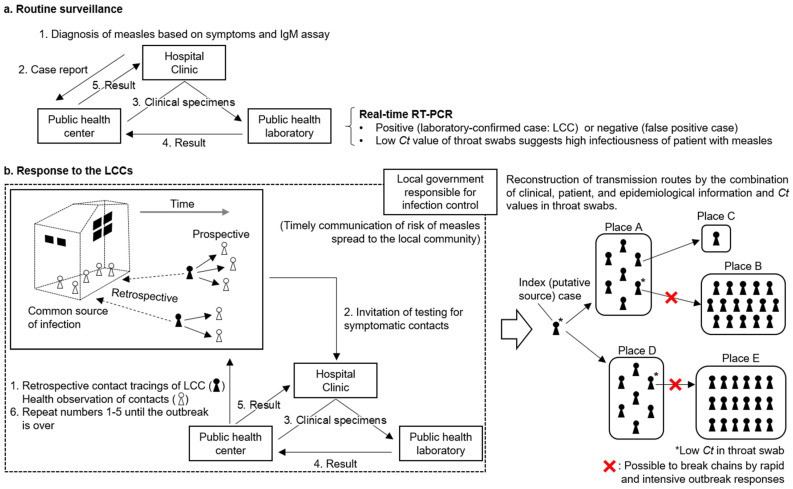
Assumed model for breaking the chain of measles transmission through the cooperation of clinical, epidemiologic, and laboratory diagnostic activities in the subnational regions of measles-eliminated countries. (**a**) In routine surveillance, quality-assured real-time RT-PCR contributes to the rapid and accurate diagnosis of acute measles cases. (**b**) Once a laboratory-confirmed case is detected, contacts and common sources of infection should be identified by retrospective and prospective contact tracings. The local government should timely communicate the risk of measles spreading to the local community until the end of measles transmission. Real-time RT-PCR should be conducted on symptomatic contacts as soon as possible. When a laboratory-confirmed case is estimated as a super-spreader candidate from the result of low *Ct* value (i.e., high viral load) in a throat swab, intensive outbreak responses to the contacts (e.g., strengthening quarantine, and testing in the asymptomatic phase) are expected to break the chain of transmission. These rapid and intensive responses would lead to the early end of measles outbreaks.

This subsection shows the basic public health responses of measles. The physician who diagnoses a patient with measles clinically requests an IgM assay for MeV and reports the case to a public health center (Figure 3a). Clinical symptoms, clinical course, vaccination history, and travel history to measles-endemic countries should also be shared. Public health centers obtain clinical specimens (e.g., whole-blood, throat swab, and urine) of patients and transport them to public health laboratories (i.e., 85 prefectural/municipal public health institutes in Japan) as soon as possible. Public health laboratories immediately perform real-time RT-PCR [17]. Technically, results can be obtained up to the next day, because real-time RT-PCR can be completed within a few hours. The test results must be shared rapidly with the public health center and the primary physician.

When a real-time RT-PCR-positive case (hereafter referred to as a laboratory-confirmed case) is detected, public health centers immediately initiate public health responses to break the chain of transmission, as described in the next subsection. Information on case detection should be conveyed to hospitals/clinics throughout the region to facilitate the accurate diagnosis of measles, as this is considered a rare infectious disease in measles-eliminated countries. This information might be shared with facilities, such as preschools and elementary schools, to encourage the administration of two doses of routine vaccinations for protecting children from directly or indirectly contracting this highly contagious infectious disease [1]. Furthermore, the department of local government responsible for infection control (e.g., prefectural office in Japan) communicates the risk of measles spreading to the local community, even if there is only knowledge of one measles case. The incubation period for measles is approximately 10–14 days [1]. Rapid diagnosis of an initial case, communication of risk to physicians and the community, and rapid public health responses by public health centers are key to preventing the spread of measles.

In contrast, if a clinically diagnosed case is negative through real-time RT-PCR and is not considered a patient with measles, the notification will be withdrawn. It is worth noting that an IgM assay sometimes shows a false-positive reaction [16], especially in measles elimination settings. Specifically, nonspecific reactions, the interference of rheumatoid factors, or viral infections caused by human parvovirus, rubella, and human herpesvirus 6 can cause false-positive reactions [16]. A surveillance study in Japan showed that 3972 (79.3%) of 5011 cases reported by clinical diagnosis and/or positive for IgM during 2018–2021 were confirmed to not be measles, mainly due to negative results of real-time RT-PCR [32]. This suggests that real-time RT-PCR plays a crucial role in detecting measles cases in countries in which measles has been eliminated.

#### 3.2.2. Response to Laboratory Confirmed Cases

This subsection shows responses to laboratory-confirmed cases, some of which will progress to outbreak responses. When a public health center detects laboratory-confirmed cases, it is necessary to identify contacts as well as common sources of infection by retrospective and prospective contact tracings (Figure 3b). This approach is in common with the “cluster-based approach” used to counter COVID-19 in Japan [5]. Thereafter, the public health center should evaluate the susceptibility of these contacts. Post-exposure prophylaxis should be administered within 72 h of the first exposure to susceptible contacts [33]. Furthermore, public health centers should conduct health observations of contacts during a certain period, for example 5–14 days after the final contact. Close contacts should be requested to self-quarantine. Contacts showing any symptoms should be immediately sent to hospitals/clinics to obtain specimens for real-time RT-PCR (Figure 3b).

Real-time RT-PCR has an advantage in that can estimate the infectiousness of patients with measles [10,11]. Specifically, low *Ct* values (i.e., high viral load) in throat swab specimens suggest high infectiousness in patients with measles. Although the cut-off value of low *Ct* is required to be defined through the accumulation of data from measles outbreaks hereafter, *Ct* values ≤ 25 in throat swabs might be a rough standard of high infectiousness [10,31]. The effectiveness of measles-containing vaccines in preventing measles infection indicates that vaccination history could be an indicator of the infectiousness of patients with measles [1]. However, the human memory and records are ambiguous. Furthermore, primary or secondary vaccine failures may occur [6,12]. Given these circumstances, the *Ct* values of throat swabs are likely to be an objective indicator of the infectiousness of patients with measles.

Superspreading, where relatively few individuals cause a large number of secondary cases, is an important phenomenon in the transmission of infectious diseases [13]. Considering that this phenomenon has been the driving force of large outbreaks in measles-eliminated countries [9,10,11], the detection of super-spreader candidates who show low *Ct* values in throat swabs would be useful for efficiently preventing the spread of measles [10]. In actual public health responses, public health centers should conduct intensive surveillance of super-spreader candidates. For example, self-quarantine should be strongly urged for contacts of potential super-spreaders to prevent further measles transmission. In addition, it might be possible to apply real-time RT-PCR to close contacts of potential super-spreaders in the asymptomatic phase (e.g., 7 days after the initial contact). If a super-spreader candidate has visited a facility used by an unspecified number of people (e.g., shopping malls and airport [12]), the local government should communicate the name of the facility and the date and time of possible infection to the local community. Through these efficient public health responses, it is possible to break the chain of measles transmission (Figure 3b).

Once sample quality and inspection accuracy are ensured (Table 1), a high *Ct* value or negative throat swab test result suggests a low infectiousness of the patient. If a person who has received two doses of a measles-containing vaccine comes into contact with a patient with low infectiousness, quarantining of the contact might no longer be needed. In this way, mitigation leads to efficient outbreak responses in public health centers with limited human resources. However, since measles is a highly contagious disease [1], mitigation should be decided carefully based on the results of further studies.

*Ct* values also provide useful information for clinicians. Specifically, *Ct* values have the advantage of estimating the following clinical course at the time of the onset of initial symptoms. We propose the collection of specimens from patients showing initial symptoms as early as possible. Therefore, laboratory-confirmed cases before the appearance of a rash could be subdivided into modified measles and the prodromal stage of measles with typical symptoms (e.g., fever, rash, and at least one of either cough, coryza, or conjunctivitis [1]). Since patients in the prodromal stage of typical measles show low *Ct* values in the specimens of PBMCs, throat swabs, and urine at the initial symptomatic stage [10], *Ct* values could be used as an indicator to identify patients who may develop typical measles. Given that measles is a potentially fatal illness [1], careful symptomatic treatment is recommended for patients with low *Ct* values even if they show mild symptoms at the time of diagnosis.

Rapid and intensive public health responses using *Ct* values of real-time RT-PCR with the cooperation of hospitals/clinics, public health centers, public health laboratories, and local governments would lead to the early end of a measles outbreak in a subnational region. It is expected that these responses in each subnational region will maintain the status of measles elimination at the national level.

### 3.3. Other Potential Laboratory Diagnostic Methods

Previous serological studies showed that an IgG avidity assay has the potential to provide information on the infectiousness and breakthrough infection of patients with measles [6,11,34]. An IgG avidity assay measures the antigen-binding avidity of IgG antibodies for MeV by removing low-avidity antibodies produced at an early stage of a primary infection [6,12]. Regarding the effectiveness of IgG avidity assays, an outbreak study indicated that acute sera in the breakthrough cases showed high avidity, whereas those in the naïve cases showed no or low avidity [11].

To strengthen the national and subnational capacity for outbreak preparedness and response, the investigation of timely and effective laboratory diagnostic methods through the analyses of measles outbreaks needs to continue hereafter [31].

### 3.4. Limitations

This study has several potential limitations. First, there was only one example of a real-world investigation that supported our potential strategy so far. An outbreak study showed that five patients, whose throat swabs showed *Ct* values in the range of 17–22, transmitted measles to their contacts [11]. Further investigations are necessary to support our strategy. Second, since we assumed outbreaks in areas with high vaccination coverage, our strategy is inapplicable to outbreaks in groups with low vaccination coverage such as religious groups who are hesitant to use vaccines [7]. Every patient in these outbreaks should be treated as a potential super-spreader, given the transmissibility of MeV [1,14]. Third, our strategy allows for the estimation of naïve and breakthrough cases by combining vaccination history and *Ct* values; however, absolute conclusions are difficult to obtain. The classification of naïve (i.e., high infectiousness) and breakthrough (i.e., low infectiousness) cases is important for outbreak responses [11,12]. In this regard, the combination of the following two laboratory test results of *Ct* values and IgG avidities might be used for the classification (Table 2). Further systematic surveillance would be performed if this hypothesis was proven. Fourth, we assumed that the high infectiousness of patients with measles could be estimated when throat swabs collected at the initial symptomatic stage showed low *Ct* values. This proposal is supported by the results of an experimental model, in which the viral load in naïve monkeys challenged with pathogenic MeV rose sharply within a few days [35]. To understand the appropriate period for sample collection, the kinetics of the viral load in the human respiratory tract should be elucidated in future studies. Fifth, we propose that throat swabs could be used to estimate infectiousness since MeV is shed from the respiratory tract. In contrast, previous studies have shown that *Ct* values of urine and PBMCs are significantly lower in the naïve cases than in the breakthrough cases [11,12]. The interpretation of “low *Ct* values” by combining results from multiple specimen types should be further investigated. The final limitation is that we used *Ct* values as an indicator of the viral load often used in infectious disease epidemiology [10,11,30]. However, the *Ct* value is a tentative measure of viral load. Ideally, genome copies should be calculated to accurately evaluate the viral load in each specimen (Table 1).

**Table 2:** Classification of the naïve and breakthrough measles cases in specimens at the initial symptomatic stage [11,12,34].

		*Ct* Value in Throat Swab	
		Low ^1^	High or Not Detected
IgG avidity ^2^	Low	Naïve	Improbable
	High	Debatable	Breakthrough

^1^ A cut-off value has not been defined, but a previous study showed values ≤ 25 to be low *Ct* values [10]. ^2^ Acute sera in breakthrough cases show high avidities (>60%) by their immune memories for MeV [6,11,12].

The resolution of these practical issues will lead to effective and efficient outbreak responses by public health centers with limited human resources. Furthermore, it might provide knowledge to save numerous costs against measles outbreaks [10].

## 4. Conclusions

A nationwide vaccination policy is the most important factor for achieving measles elimination. In measles-eliminated countries, it is necessary to sustain vaccination coverage in more than 95% of the population as well as to conduct timely and effective public health responses to measles outbreaks through the cooperation of hospitals/clinics, public health centers, public health laboratories, and the local government [31]. The high basic reproduction number of measles (R_0_ = 12–18) suggests that patients with high infectiousness can easily become super-spreaders [1,10,11,13]. This opinion proposes a possible strategy of rapid and intensive responses to counter measles outbreaks caused by super-spreader candidates showing low *Ct* values in throat swabs. Our strategy would make it possible to effectively prevent further measles transmission, thereby leading to the early termination of measles outbreaks. Under the concern of a worldwide re-emergence of measles [1,3], a wider discussion of this strategy is desirable hereafter to accelerate the use of laboratory test results in outbreak responses for measles.

## Figures and Tables

**Table 1:** Requirements for accurate real-time RT-PCR without false-negative results [1,10,11,12,29].

Category	Subcategory	Content
Sample quality	Specimen collection	Collect throat (or nasopharyngeal) swabs using a synthetic fiber swab. Serum is unsuitable for the breakthrough cases.
	Multiple specimens	Collect multiple specimens (e.g., throat swab, whole-blood, and urine). PBMCs, extracted from whole-blood, and throat swabs show a high positive rate among the breakthrough cases.
	Collection timing	Collect specimens at the time of initial symptoms. Ideally collect specimens within 7 days of rash onset.
	Transport conditions	Require cold chain and rapid transportation using a viral transport media to minimize the fragmentation of viral RNA.
	Multiple tests	Collect specimens again when initial testing is negative, but measles is strongly suspected.
Inspection accuracy	Accuracy assurance	Prepare standard operating procedure for specimen storage, specimen pretreatment, RNA extraction, real-time RT-PCR, and result determination. Implement the internal quality control and the external quality assessment. Implement regular equipment maintenance.
	Negative control	Confirm negative result in negative control in every test to confirm absence of laboratory cross-contamination.
	Positive control	Confirm the detection of measles virus standard RNA with low copy numbers (e.g., 50 copies) in every test to ensure the inspection accuracy.
	Calibration curve	Ideally obtain a calibration curve using serially diluted standard RNA samples in every test to calculate genome copies of MeV.
